# To Have the Best Interest at Heart: Analyzing the Match Between Laypersons’ Interests and Publication Activity in Psychology

**DOI:** 10.3389/fpsyg.2022.899430

**Published:** 2022-06-02

**Authors:** Mark Jonas, André Bittermann, Anita Chasiotis, Tom Rosman

**Affiliations:** Leibniz Institute for Psychology (ZPID), Trier, Germany

**Keywords:** topic interest, research topics, publication trends, literature analysis, lay summaries, science communication, research syntheses

## Abstract

There is a growing public interest in science and, by extension, in psychology, and human behavior. Yet, detailed investigations on whether academic psychological research activity matches lay interests are still scarce. In addition, while lay-friendly communication of research findings becomes continually more important, it is unclear which subfields of psychological research are particularly interesting to laypeople. To address these research gaps, we carried out an explorative study of psychological literature included in two large reference databases, one with a German (*PSYNDEX*) and one with an international (*PsycInfo*) scope. The years of 2018–2020 were scanned for articles belonging to one of 20 topic areas assessed as most interesting by lay participants in a previous study. We determined and compared the share of empirical research and research syntheses for each topic area and database and computed rank correlations between lay interest and academic publication volume. Results suggest a positive relationship between lay interest and academic publication activity specifically for research syntheses. Additionally, topic areas associated with clinical psychology offered a large share of research syntheses, while other topic areas such as “Psychodynamics” or “Industrial & Organizational Psychology” encompassed a smaller share of syntheses. Finally, we outline perspectives for long-term monitoring of psychology-related lay interests. Thus, the present study connects academic activity with the public interest in psychology by identifying and quantifying research syntheses for topics garnering the most lay interest.

## Introduction

People often gravitate toward psychological topics during conversations. Questions like “How can someone come to believe that the COVID-19 pandemic is a hoax?” “Why does he/she dismiss man-made climate change when there is clear scientific evidence?” or “I lately feel overwhelmed and incredibly sad, what should I do?” are but a few examples. Seeking answers for the behavior of others or our own emotional experiences seems to be of fundamental interest to us as humans. And indeed, such an interest in psychological questions has emerged repeatedly. For example, McPhetres (2019) was able to demonstrate that topics from the fields of psychology, social science or health were among the most upvoted categories on Reddit.

In line with such public interest in science and psychology, science communication becomes increasingly important. Multiple reasons for this can be identified: First, the current era of “big literature” (Nunez-Mir et al., 2015), characterized by an increasing number of scientists and scientific publications (Wang and Barabási, 2021) creates difficulties for scientists and lay readers alike when trying to gauge the scope of scientific evidence. Second, it can often be challenging to gain access to said evidence. The scientific jargon in publications has become increasingly difficult to read in recent years (Plavén-Sigray et al., 2017), paywalls still limit access to journal articles (Martín-Martín et al., 2018; Piwowar et al., 2018), and complex methodologies like extensive literature reviews or meta-analyses can topple comprehension. Especially for lay readers, this may make it cumbersome to gain access to relevant research findings. Finally, a lag usually exists between the first emergence of pressing societal issues (e.g., climate change denial; COVID-19 measures and their consequences for mental health; migration and immigration) and the publication of empirically based popular science works. In the meantime, interested readers may have to rely on regular internet searches as an information source. While certainly a fast and popular approach (Nielsen DCM, 2019), this may also expose readers to statements of unclear scientific quality and validity. Lay readers may thus be at an increased risk of drawing on logical fallacies (e.g., cherry-picking, goal posting, motivated reasoning, see Hoofnagle and Hoofnagle, 2007), and fail to identify problematic research practices employed by some scientists (e.g., p-hacking, harking). When worse comes to worse, this could fuel the spread of misinformation and diminish trust in science and scientists (Head et al., 2015; Chambers, 2019; Murphy and Aguinis, 2019).

It appears vital to tackle these difficulties. A promising step and possible gateway toward greater involvement of laypeople in psychological research could be to offer plain language summaries (PLS; McIlwain et al., 2013; Langendam et al., 2013) to lay readers. PLS aim to maximize the accessibility of research findings by avoiding scientific jargon and complicated wording, briefly emphasizing key points, and describing the overall quality of evidence. While up until now mostly used in the medical field, PLS have recently also gained traction in psychology (Stricker et al., 2020), specifically in the context of meta-analyses (Kerwer et al., 2021a). A reason for this can be found in the benefits that meta-analyses offer compared to individual studies: Since they usually include standardized effect sizes synthesized from multiple empirical studies, their findings are less prone to distortions and offer a greater degree of generalizability. As a result, PLS of psychological meta-analyses may function as an attractive and reliable source for laypersons interested in specific psychological topics. The implementation of such a PLS-based approach in psychology may be especially fruitful for topic areas that already pique laypeople’s interest. In contrast, even perfectly written PLS are prone to receive only limited attention at best if they focus on topics deemed uninteresting or irrelevant. As of now, however, little research exists in this field, and it is unclear which psychological topic areas specifically attract laypeople. Further investigations are thus needed.

**FIGURE 1:**
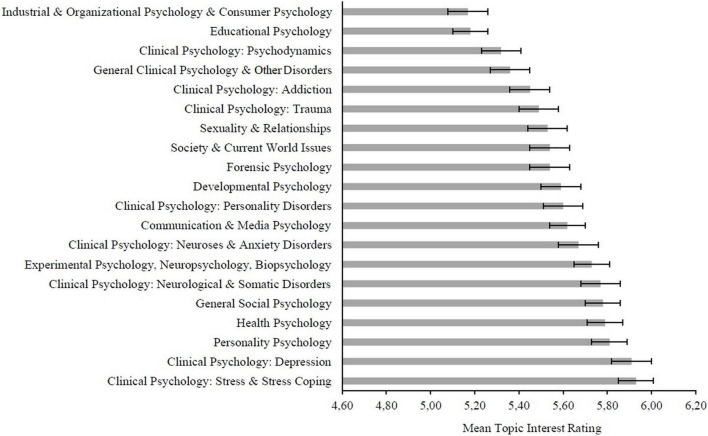
Overview of the mean interest ratings of study I and II participants for all 20 topic categories with 95% confidence intervals (reproduced with permission from Benz et al., 2021).

To shed some light on the interests of laypersons in the field of psychology and to further differentiate between topics of high and low interest, we recently conducted two large-scale user surveys in the context of a project called “Plain Language Summaries for Psychological Meta-Analyses” (Project PLan Psy; Kerwer et al., 2021b). In a first online study, 2,038 participants from a general population sample and without a background in psychology (i.e., not currently enrolled or holding a degree in psychology) were, in addition to questions regarding different PLS, asked about their interest in psychology topics (“About which psychology topics would you like to know more?”). Up to three different topics could be named in a free-text format, which were subsequently coded by two independent raters *via* a qualitative coding scheme. Thereafter, they were synthesized into 20 overall interest categories such as “Developmental Psychology” or “Clinical Psychology: Depression” (see Supplementary Material 1 for an overview). In the next step, the 20 topic categories were reexamined in the context of a second online study (Kerwer et al., 2021b) to confirm their relevance and their external validity. Once again, a general population sample of 2,083 participants with diverse educational attainment levels, excluding participants with a background in psychology, was recruited. Participants once more completed a user survey and were asked about their interest in the 20 psychological categories obtained from Study I (“How strongly are you interested in the topic …?”). They answered this question on a Likert-type scale ranging from 1 to 8 (1 = “not interested at all,” 8 = “very interested”). Results indicated an overall high interest for each of the categories, with ratings ranging from 5.17 (*SD* = 2.10) for “Industrial & Organizational Psychology & Consumer Psychology” to 5.93 (*SD* = 1.93) for “Clinical Psychology: Stress & Stress Coping.” These results imply that the extracted topic categories are indeed of substantial interest for individuals from a general population sample (see Figure 1 for a more comprehensive overview over participants’ interest ratings by topic category).

The findings of these two studies offer some insight into the nature of topics that currently interest the public, yet warrant some further clarification. On the one hand, while some of the most common topic categories of psychology could be identified, fields of applied psychology—for example engineering psychology, traffic psychology or sport psychology—were not mentioned by laypeople. This stands at odds with considerable research and publication activities in these fields^1^. On the other hand, it is still unclear how well research and publication activities cover topic categories deemed as highly interesting by laypersons. Therefore, it seems warranted to take a closer look at the relationship between the public’s interest in psychological topics and their actual coverage by research efforts. In addition, the amount of available synthesized empirical evidence (i.e., meta-analyses and systematic reviews) may vary widely between psychological topics, resulting in implications for the creation of PLS. Especially topic areas with a rich base of synthesized evidence deemed interesting by laypersons may be adequate starting grounds for more wide-scaled PLS efforts. It could thus prove beneficial to examine differences in available meta-analytic evidence more thoroughly. A final point concerns a specific sub-topic of the 20 topic categories, namely “Society & Current World Issues.” In Study I, the category served as a way to include participants’ interest in current societal events such as the COVID-19 pandemic, migration and immigration, or environmental issues. During the Study II validation process, this category ranked 13th out of 20, with a participant mean interest rating of 5.54 (*SD* = 2.04, range = 1–8). This supports the notion that, above and beyond “core topics,” psychological questions associated with current societal events also evoke the attention and interest of the public. And indeed, nationally and internationally renowned professional organizations such as the German Science and Humanities Council (Wissenschaftsrat, 2018), the German Society for Psychology (Antoni, 2019; Spinath, 2021), the American Psychological Association (American Psychological Association [APA], 2019), or the British Psychological Society (British Psychological Society [BPS], 2015) have recently advocated for a more proactive and transparent position of psychological research toward current societal and political issues. Fortunately, there is reason to assume that research activity in psychology is indeed permeable to current societal trends. For instance, public interest in topics such as migration, climate change, or digitalization over the last few years is mirrored by publication trends in psychology (Bittermann and Klos, 2019; Bittermann, 2021). Furthermore, the psychological research community is quick to pick up developments such as COVID-19 or climate change *via* online posts on microblogs such as Twitter (Bittermann et al., 2021). Still, a current, up-to-date comparison of laypersons’ topic interests with psychological research activity could further strengthen the position of psychology as a scientific domain right on the pulse of time.

The present explorative study therefore aims to compare the 20 topics identified and validated in the two prior studies described above to the academic research and publication activity in psychology. We thus intend to answer the following research questions (RQ):

*RQ1*: To what extent are laypersons’ topics of interest currently addressed in psychological research?

*RQ2*: What is the share of research syntheses by each topic, as a sound evidence basis for plain language summaries (PLS)?

To examine these questions, we queried psychological literature databases in order to assess the publication volume by topic. Importantly, the respective share of current research syntheses was determined, as they play a key role in communicating reliable psychological evidence (i.e., summarizing the current state of knowledge). Enriching such syntheses with PLS can support the dissemination of scientific results of particular interest to the public: Up-to-date and summarized psychological knowledge that is easy to comprehend can empower the public to access scientific knowledge directly, without waiting for popular science works to be published or relying on information from questionable internet sources. Hence, the present study contributes to bridging the gap between academia and the public by identifying and quantifying research syntheses of topics associated with high layperson interest.

## Materials and Methods

For laypersons interested in current psychological evidence, up-to-dateness of research is crucial. Shojania et al. (2007) reported a median duration of 5.5 years for systematic reviews to become outdated. Given the exponential growth of scientific publications in recent years (Wang and Barabási, 2021), we included publications from the last 3 years preceding the 2021 survey on laypersons’ interests (Kerwer et al., 2021b), i.e., 2018–2020. To find matching psychology publications for laypersons’ interest topics, we queried^2^ the psychological reference databases *PsycInfo* (produced by the American Psychological Association; APA) and—as the laypersons’ topics stem from a German sample—*PSYNDEX* (produced by Leibniz Institute for Psychology, Germany). With a separate analysis of *PsycInfo* as an international database (with a strong Anglo-American focus; Krampen, 2016) and *PSYNDEX* as a database for research from the German-speaking countries, it can be determined whether a potential match between laypersons’ interest topics and research topics is regionally limited or generalizable. For generating search strings, we consulted topic notes in the PLan Psy report (Benz et al., 2021) and used classification codes^3^ along with terms of the APA’s *Thesaurus of Psychological Index Terms* (Tuleya, 2007) and *PSYNDEX Terms* (ZPID, 2016). In addition, thesaurus terms as well as topic terms not covered by the thesaurus (e.g., “hypervigilance”) were searched for in titles, ensuring that these terms reflected the publication’s main subject. For each topic and database, the absolute frequency of records as well as the relative frequency regarding the total volume of publications in 2018–2020 was compared to the 20 categories stemming from laypersons’ interest ratings. For example, our previous studies indicated that laypersons were most interested in the research fields of “Clinical Psychology: Stress & Stress Coping;” “Clinical Psychology: Depression;” “Personality Psychology;” “Health Psychology” and “General Social Psychology” (a complete overview of all 20 topic categories can be found in Figure 1). In the present study, comparing previously recorded laypersons’ interest ratings with academic publication activity allowed us to descriptively assess the general overlap or mismatch between academic research topics and laypersons’ interests (RQ1). In a final step, we filtered the results for meta-analyses and systematic reviews and manually screened them for eligibility. For instance, systematic reviews addressing methodological issues in social psychology might be indexed by the databases as social psychology publications and thus included in our search results. However, the laypersons in the study of Kerwer et al. (2021b) did not mention methods, but rather social interaction, social perception, stereotyping etc. Hence, in this step of manually screening research syntheses for eligibility, we removed all records unintended by the laypersons (according to topic definitions by Benz et al., 2021). Having checked the match between the research syntheses with the laypersons’ interests, we calculated relative frequencies of all meta-analyses and systematic reviews from 2018 to 2020. This allowed us to compare topics with different publication volumes. The share of available research syntheses was compared to assess the existing quantity of summarizing research available for communication to laypersons *via* PLS (RQ2). During this process, we aimed to assess if small percentages of research syntheses could be traced back to a topic’s inherent characteristics (e.g., low shares of empirical research due to the recent emergence of a topic) and if a shift toward publishing more research syntheses may be desirable.

## Results

Overall, the literature search resulted in a total of 505,141 *PsycInfo*- and 38,653 *PSYNDEX*-articles published between 2018 and 2020. Of these, a total of 349,299 (69.15%) for *PsycInfo* and 21,211 (54.88%) for *PSYNDEX* were empirical research publications. Finally, 11,290 (2.24%) publications for *PsycInfo* and 787 (2.04%) publications for *PSYNDEX* qualified as research syntheses matching laypersons’ interests. The manual inspection for eligibility of research syntheses led to the exclusion of 4,030 publications (26.31% of 15,320 research syntheses matching the search query) in *PsycInfo* and 118 Publications (13.04% of 905) in *PSYNDEX*. A complete overview of the amount and percentages of publication types for each interest category can be found in Table 1.

**TABLE 1:** Laypersons’ topics of interest and related records in psychological databases.

	PsycInfo				PSYNDEX		
Topic	Interest *M* (*SD*)	Publications *n* (%*)	Empirical Research *n* (%**)	Research Syntheses *n* (%**)	Publications *n* (%*)	Empirical Research *n* (%**)	Research Syntheses *n* (%**)
Clinical Psychology: Stress & Stress Coping	5.93 (1.93)	10,966 (2.06%)	8,414 (76.73%)	153 (1.40%)	1,238 (3.62%)	746 (60.26%)	16 (1.29%)
Clinical Psychology: Depression	5.91 (2.01)	21,001 (3.94%)	14,731 (70.14%)	1,067 (5.08%)	1,493 (4.36%)	908 (60.82%)	75 (5.02%)
Personality Psychology	5.81 (1.92)	10,485 (1.96%)	7,326 (69.87%)	93 (0.89%)	1,705 (4.98%)	1,155 (67.74%)	54 (3.17%)
Health Psychology	5.79 (1.94)	16,565 (3.10%)	11,611 (70.09%)	646 (3.90%)	1,379 (4.03%)	719 (52.14%)	47 (3.41%)
General Social Psychology	5.78 (1.88)	12,659 (2.37%)	8,691 (68.65%)	89 (0.70%)	1,802 (5.26%)	1,395 (77.41%)	37 (2.05%)
Clinical Psychology: Neurological & Somatic Disorders	5.77 (1.97)	45,440 (8.52%)	32,036 (70.50%)	590 (4.90%)	5,489 (16.03%)	4,475 (81.53%)	93 (3.18%)
Experimental Psychology, Neuorpsychology, Biopsychology	5.73 (1.93)	54,655 (10.24%)	43,925 (80.37%)	590 (1.08%)	5,489 (16.03%)	4,475 (81.53%)	93 (1.69%)
Clinical Psychology: Neuroses & Anxiety Disorder	5.67 (2.07)	10,813 (2.03%)	8,821 (81.58%)	404 (3.74%)	1,195 (3.49%)	660 (55.23%)	40 (3.35%)
Communication & Media Psychology	5.62 (1.96)	12,345 (2.31%)	8,811 (71.37%)	121 (0.98%)	1,300 (3.80%)	682 (52.46%)	12 (0.92%)
Clinical Psychology: Personality Disorders	5.60 (1.99)	1,543 (0.29%)	931 (60.43%)	46 (2.98%)	475 (1.39%)	206 (43.37%)	7 (1.47%)
Developmental Psychology	5.59 (1.99)	27,742 (5.20%)	20,439 (73.68%)	402 (1.45%)	2,919 (8.52 %)	1,882 (64.47%)	46 (1.58%)
Forensic Psychology	5.54 (2.02)	11,348 (2.13%)	7,041 (62.05%)	221 (1.95%)	921 (2.69%)	387 (42.02%)	7 (0.76%)
**Society & Current World Issues**							
Coronavirus	5.54 (2.02)	4,269 (0.80%)	1,035 (24.24%)	35 (0.82%)	225 (0.66%)	29 (12.89%)	1 (0.44%)
Lockdown & Quarantine	5.54 (2.04)	309 (0.06%)	120 (38.83%)	1 (0.32%)	25 (0.07%)	3 (12.00%)	0 (0.00%)
Climate Change	5.54 (2.04)	744 (0.14%)	461 (61.96%)	10 (1.34%)	73 (0.21%)	28 (38.36%)	2 (2.74%)
Migration	5.54 (2.04)	7,596 (1.42%)	5,200 (68.46%)	105 (1.38%)	908 (2.65%)	400 (44.05%)	11 (1.21%)
Political Crises	5.54 (2.04)	32 (0.01%)	17 (53.13%)	0 (0.00%)	4 (0.01%)	2 (50.00%)	0 (0.00%)

Sexuality & Relationships	5.53 (2.05)	8,239 (1.54%)	5,307 (64.41%)	77 (0.93%)	1,147 (3.35%)	588 (51.26%)	15 (1.31%)
Clinical Psychology: Trauma	5.49 (2.02)	12,640 (2.37%)	7,627 (60.43%)	358 (2.83%)	1,628 (4.75%)	655 (40.23%)	29 (1.73%)
Clinical Psychology: Addiction	5.45 (2.07)	12,485 (2.34%)	9,030 (72.33%)	495 (3.69%)	765 (2.23%)	390 (50.98%)	19 (2.48%)
General Clinical Psychology & Other Disorders	5.36 (2.06)	122,282 (22.92%)	73,352 (59.99%)	2,739 (2.24%)	5,432 (15.84%)	1,791 (33.03%)	109 (2.01%)
Clinical Psychology: Psychodynamics	5.32 (2.12)	4,004 (0.75%)	171 (4.72%)	5 (0.12%)	1,196 (3.49%)	61 (5.10%)	2 (0.17%)
Educational Psychology	5.18 (2.05)	58,430 (10.95%)	45,315 (77.55%)	751 (1.29%)	2,397 (7.00%)	1,412 (58.91%)	46 (1.92%)
Industrial & Organizational Psychology & Consumer Psychology	5.17 (2.10)	38,549 (7.22%)	28,887 (74.94%)	654 (1.70%)	2,522 (7.37%)	1,144 (45.36%)	42 (1.67%)

The topics are sorted by interest ratings, ranging from 1 = “not interested at all” to 8 = “very interested.” Databases were queried in November 2021. The research syntheses included were meta-analyses and systematic reviews, screened manually for eligibility. Topics and interest ratings from Project PLan Psy (Benz et al., 2021). *Related to all records in the database (2018–2020). **Related to all publications within a topic.

To first address the research question of the current coverage of laypersons’ topics of interest in psychological research (RQ1), Kendall’s τ coefficients for the relation between interest ratings and database publication numbers were computed due to lack of normality and the small number of observations. The overall association between laypersons’ interest and the respective publication volume was τ*_*b*_* = 0.02, *p* = 0.92, 95% CI = [−0.27, 0.30] for *PsycInfo* and τ*_*b*_* = 0.05, *p* = 0.73, 95% CI = [−0.21, 0.32] for *PSYNDEX*. For research syntheses, this association was τ*_*b*_* = 0.14, *p* = 0.36, 95% CI = [−0.16, 0.44] for *PsycInfo* and τ*_*b*_* = 0.27, *p* = 0.08, 95% CI = [−0.01, 0.54] for *PSYNDEX*, indicating a small (however non-significant) positive association between laypersons’ interest and share of published research syntheses: In both databases, the (with regard to interest) high ranking topics “Clinical Psychology: Depression,” “Health Psychology,” and “Clinical Psychology: Neurological & Somatic Disorders” had larger and comparable shares of research syntheses. In *PSYNDEX*, this also applied to the topics “Personality Psychology” and “General Social Psychology” (thus yielding a larger τ*_*b*_* coefficient). For the topic rated as most interesting (i.e., “Clinical Psychology: Stress & Stress Coping”), the share of research syntheses was rather small and below average in both databases (1.40% in *PsycInfo* vs. 1.29% in *PSYNDEX*; with *M*_*PsycInfo*_ = 2.24 % and *M*_*PSYNDEX*_ = 2.04%).

Concerning the research question focused on the share of research syntheses by topic (RQ2), Table 1 shows that their relative frequencies ranged from 0 to 5%. Taking a descriptive approach, we broadly summarize our results as follows: First, topics with a focus on clinical or health psychology had a comparatively large share of research syntheses in both databases. We observed the largest shares for the topic “Clinical Psychology: Depression” (5.08% in *PsycInfo* vs. 5.02% in *PSYNDEX*). Also ranking high in both databases were the topics “Clinical Psychology: Neurological & Somatic Disorders” (4.90 vs. 3.18%), “Health Psychology” (3.90 vs. 3.41%), and “Clinical Psychology: Neuroses & Anxiety Disorders” (3.74 vs. 3.35%). A notable exception was “Clinical Psychology: Psychodynamics,” with low shares for both empirical research (4.27% in *PsycInfo*, 5.10% in *PSYNDEX*) and research syntheses (0.12% in *PsycInfo*, 0.17% in *PSYNDEX*). Second, psychology topics outside the clinical domain generally displayed a high share of empirical research, but a considerably lower share of research syntheses. For instance, a large share of empirical work was available for “Experimental Psychology, Neuropsychology, Biopsychology” (*PsycInfo*: 80.37%, *PSYNDEX* = 81.53%) and “Educational Psychology” (*PsycInfo*: 77.55%; *PSYNDEX* = 58.91%), but the share of research syntheses was more limited (“Experimental Psychology, Neuropsychology, Biopsychology:” *PsycInfo* = 1.08%, *PSYNDEX* = 1.69%; “Educational Psychology:” *PsycInfo* = 1.29%, *PSYNDEX* = 1.92%). As a descriptive tendency, the share of accessible research syntheses was slightly higher in *PSYNDEX* for these topics. And third, only comparatively little empirical research and almost no research syntheses were available for research topics associated with “Society & Current World Issues.” Therefore, concluding statements about differences in research syntheses volumes are not feasible. However, when descriptively examining the published empirical research, the publication shares for longer-running current topics such as “Climate Change” (*PsycInfo*: 1.34%, *PSYNDEX*: 2.74 %) or “Migration” (*PsycInfo*: 1.38%, *PSYNDEX*: 1.21%) were descriptively larger compared to the very recent topic “Coronavirus” (*PsycInfo*: 0.82%, *PSYNDEX*: 0.44%).

## Discussion

The present exploratory study aimed to shed light upon the match between laypersons’ psychological interests and academic researchers’ publication activity in two large-scale psychology databases between 2018 and 2020. A substantial number of publications could be identified within an international (*PsycInfo*) and a German (*PSYNDEX*) database for all 20 topic categories previously rated as interesting (Benz et al., 2021). Regarding our initial question about the overlap between overall publication volume per topic category and laypersons’ interest (RQ1), no positive relationship emerged. However, we observed a tendency for a positive relationship within the subfield of research syntheses, i.e., a higher number of syntheses was available in topic areas with higher lay interest. This was particularly the case in the *PSYNDEX* database for literature from German-speaking countries, even though this association only reached marginal significance. Nevertheless, for most topics of laypersons’ interest, a substantial number of research syntheses has been published in the last 3 years preceding the interest survey (i.e., 2018–2020). This was particularly true for topics related to clinical psychology, which make up the bulk of the interest areas.

Taking these findings together, we preliminarily conclude that research syntheses publications (i.e. systematic reviews or meta-analyses) indeed provide a certain match with lay reader interests and may serve as a promising and sound basis to communicate scientific evidence. However, this conclusion should be taken with a grain of salt. More precisely, the 20 proposed topic categories display varying levels of content heterogeneity (e.g., “General Social Psychology” vs. specific subtopics of clinical psychology such as “Clinical Psychology: Depression”). These varying levels of specificity may contribute to a lower number of research syntheses for more heterogeneous topics despite ample availability of empirical research works. In connection to this, certain fields (e.g., forensic psychology, political psychology) may simply be less well represented than others. Renowned fields such as clinical psychology are likely to be more prevalent in terms of university chairs, and fields with stronger economic ties such as consumer research may be more prevalent in terms of commercial research carried out by individual scientists and market research institutes. In terms of layperson interest, these fields may simply have the advantage of their interdisciplinary nature as well as the comparatively larger amount of publications when it comes to capturing attention. It should also be noted that laypersons’ interest categories are themselves heterogeneous. In order to allow for a manageable number of interest topics, some categories such as “General Clinical Psychology & Other Disorders” or “Clinical Psychology: Neurological & Somatic Disorders” were defined rather broadly. This resulted in a larger number of included studies and may again lead to a considerable body of empirical research works with a comparatively smaller number of research syntheses. Such a level of fine-grained differentiation was, however, beyond the scope of the present study. Future studies could address this issue by defining interest categories more exhaustively and distinctively. Notwithstanding, research syntheses may prove to be a promising source for directly addressing the public’s interest in psychological research insights. Considering the paramount benefits of communicating high-quality evidence from research syntheses to laypersons, this is good news: Not only may PLS of research syntheses provide their readers with the best evidence at hand, but they are also potentially likely to cover topics that are of their target group’s interest.

A second aim of the present study was to identify the share of research syntheses for each interest topic (RQ2). In summary, research topics associated with clinical psychology or health psychology displayed the highest share of research syntheses, followed by core psychology topics such as “Personality Psychology,” “Industrial & Organizational Psychology & Consumer Psychology” or “Educational Psychology.” Topics in conjunction with “Society & Current World Issues” had a tendency for a lower share of research syntheses. However, in terms of topics such as “Corona” or “Lockdown & Quarantine,” this is hardly surprising. The first known COVID-19 cases only emerged in late 2019 (Zhu et al., 2020) and a certain amount of empirical work is required for syntheses or meta-analyses to be conducted. Furthermore, time lag during data collection and publication procedures needs to be taken into account (Krampen et al., 2004). Thus, a conclusive statement about these topics cannot yet be provided. However, what remains noteworthy is that societal topics such as “Migration” or “Climate Change” descriptively exhibited a publication volume similar to that of a more traditional psychological research field like “Forensic Psychology.” A larger share of research syntheses could also be identified in these fields. This may be taken as supporting evidence for the position that psychology is indeed permeable to research topics on the mind of the public, as previously addressed in other publications (Bittermann and Klos, 2019). As noted in the introduction section, research syntheses are a more suitable base for PLS than individual studies, particularly when paired with lay reader interest in the research results they summarize. Therefore, especially topic areas such as “Clinical Psychology: Depression,” “Health Psychology” or “Clinical Psychology: Neuroses & Anxiety Disorders,” with their comparatively high share of research syntheses, offer fruitful conditions for intensifying future PLS efforts.

### Limitations and Implications for Future Research

Beyond the previous implications of topic category heterogeneity for the amount of extracted articles and the share of available research syntheses, some additional limitations apply within the context of the present study. A potential point of debate is that non-empirical articles and singular research works may also be suitable for coverage by PLS and the dissemination of knowledge to laypersons. This interjection seems especially likely from fields with a lower share of empirical publications (for instance, “Clinical Psychology: Psychodynamics”) or research syntheses (i.e., “General Social Psychology,” “Communication & Media Psychology”). Certainly, insights from these fields can prove valuable for the public. However, in line with the notion that PLS should serve as a base for informed decision-making (Nunn and Pinfield, 2014) and opinion formation, we would argue that it is crucial to first and foremost reprocess research works with a strong empirical and more bias-resistant evidence foundation into PLS (i.e., meta-analyses). Thereby, the likelihood of presenting both valid and reliable findings to the public can be maximized. Yet, a substantial challenge concerns the assessment of the overall quality of research syntheses and the primary studies they draw from. For instance, in some areas, meta-analyses may either only draw upon findings from more dated studies with lesser adherence to quality standards set by guidelines such as GRADE (Guyatt et al., 2011) or general quality assessment tools (Wedderhoff and Bosnjak, 2020), or they may show less compliance with quality criteria such as the Cochrane Guidelines for Systematic Reviews (Higgins et al., 2021). This may well result in reliability and validity differences and therefore inconsistencies between meta-analyses even within the same overall topic category. Hence, although meta-analyses display advantages over individual studies when communicating results to the public *via* PLS, they are by no means a universal remedy rendering all methodical considerations obsolete. At present, lay readers are often still tasked with making sense of such inconsistencies between syntheses works by themselves. Thoroughly taking study quality criteria into account either *via* the use of *a priori* inclusion/exclusion criteria or *a posteriori* quality coding was beyond the scope of the present explorative study, but may well be worthwhile for future studies examining the communication of synthesized evidence to the public in more detail.

From a methodological viewpoint, the fact that we focused on validating extracted literature data only for research syntheses can be criticized. The overall amount of extracted publications as well as empirical research for each category was not double-checked in terms of eligibility regarding the original intentions of laypersons. This was mainly due to pragmatic reasons, since it would have taken considerably more time and resources to validate all publication types (for instance, the largest category, “General Clinical Psychology & Other Disorders,” encompassed around 144,000 total publications for 2018–2020), and since research syntheses represented the focal point of our literature analysis. Still, the exact amount of extracted works for overall publications and empirical research may be distorted as a result. In the present study, the issue of incorrect interest category assignment did arise for research syntheses: Validating the extracted findings resulted in 26.3% of *PsycInfo* and 13.04% of *PSYNDEX* syntheses being classified as non-fitting for their initial interest category. It does not seem far-fetched to assume that this classification issue also applies to overall publications and empirical research. Thus, the exact quantity of extracted works for overall publications and empirical research should be interpreted cautiously. A future solution for problems associated with screening large-scale databases for laypersons’ interests could be to increasingly utilize machine learning tools for eligibility screening. While their implementation may be associated with initial challenges such as the need for a training data set or precisely defining stopping rules for the screening procedure itself, they have the potential to be a viable and economic tool for processing large volumes of literature (Bannach-Brown et al., 2019; Marshall and Wallace, 2019).

Additionally, it may be worthwhile to more thoroughly examine interest differences between laypeople depending on their professional background. In the two studies leading to the creation of the 20 interest categories (Kerwer et al., 2021b), laypeople with a psychology background were excluded. However, no further data on laypeople’s background were analyzed. Future studies aiming to shed light upon the overlap between the interests of laypeople coming from a particular professional field and academic publication activity in psychology could profit from carrying out analyses in more specialized samples.

As a final point, it should be noted that the interests of laypersons distilled into the 20 topic categories and utilized as building blocks for our current explorative study were probed at the beginning of 2021. As such, they provide a time-sensitive snapshot of public interest evaluations and may be heavily influenced by current events (e.g., the COVID-19 pandemic), societal trends (e.g., a growing awareness of “fake news” and conspiracy theories) or technological developments (e.g., an increasing reliance on online communication and digital media). A repeated, longitudinal measurement approach may prove beneficial for future studies in order to keep a closer tab on current public interests and their development over time. Besides repeated surveys, an unobtrusive monitoring of social media or search trends (such as Google Trends or Wikipedia page views) might be a worthwhile endeavor, especially for keeping track of emerging laypersons’ interests. Both approaches could, for instance, allow for a more nuanced perspective on the issue of low public interest in “applied” psychological fields such as engineering, traffic or sport psychology, with different implications depending on whether an increase, stagnation or reduction of interest over time is found.

## Conclusion

In this exploratory study, we examined the current match between laypersons’ interests in psychological topics and academic publication activity in psychology. We also determined the overall availability of research syntheses providing a sound evidence basis for psychological PLS. Generally, an optimistic stance seems warranted. There is ground to assume that research synthesis publication activity tends to align with the public’s interest. Furthermore, psychological research activity seems to take current events and societal debates into account. This appears especially crucial in the wake of providing up-to-date information to the public for informed decision-making. We are thus cautiously optimistic that psychology truly has the best interests of the public at heart.

## Data Availability Statement

The search query codes as well as the extracted literature data for this publication can be found in the article Supplementary Material as well as online at PsychArchives (http://dx.doi.org/10.23668/psycharchives.6678).

## Author Contributions

MJ wrote first drafts of all article sections, extracted literature data for *PsycInfo*, and was involved in validating extracted literature for *PsycInfo*. AB extracted literature data from PSYNDEX, contributed to the introduction, methods, results and discussion sections, and carried out descriptive analyses. AC provided ideas and corrections for multiple sections and wrote parts of the introduction and discussion sections. TR provided conceptual input, corrections, and proofread the manuscript. All authors read and approved the final manuscript.

## Conflict of Interest

The authors declare that the research was conducted in the absence of any commercial or financial relationships that could be construed as a potential conflict of interest.

## Publisher’s Note

All claims expressed in this article are solely those of the authors and do not necessarily represent those of their affiliated organizations, or those of the publisher, the editors and the reviewers. Any product that may be evaluated in this article, or claim that may be made by its manufacturer, is not guaranteed or endorsed by the publisher.

## Footnotes

1 A PsycInfo search for peer-reviewed empirical journal articles conducted on January 25, 2022 yielded 608 articles for “Sport Psychology” (Classification Code CC 3700), 9,156 articles for “Human Factors Engineering”/”Lifespan and Institutional Design” (CC 4010/4030) and 8,010 articles for “Transportation” (CC 4090) published between 2011 and 2021.

2 Specifically, we searched PsycInfo *via* OVID and PSYNDEX *via* PubPsych.eu. All search queries can be found in Supplementary Material 2.

3 For a full list of the classification codes used by both PsycInfo and PSYNDEX (see https://www.apa.org/pubs/databases/training/class-codes).

## Supplementary Material

Supplementary Material 1 Overview of the 20 synthesized interest topics provided by laypersons in PLan Psy Study I, their respective content and relative frequency.

Supplementary Material 2 Overview of all search query codes from PsycInfo and PSYNDEX for replication.

Supplementary Material 3 Basic bibliographic metadata of research syntheses extracted from PsycInfo after validation.

Supplementary Material 4 Basic bibliographic metadata of research syntheses extracted from PSYNDEX after validation.
