# Supplementary material for: To Have the Best Interest at Heart: Analyzing the Match Between Laypersons’ Interests and Publication Activity in Psychology
Source: Front Psychol. 2022 Jun 2;13:899430. doi: 10.3389/fpsyg.2022.899430 (PMC9201961; doi:10.3389/fpsyg.2022.899430)
Supplement: Supplementary Material 1 — Overview of the 20 synthesized interest topics provided by laypersons in PLan Psy Study I, their respective content and relative frequency. [file Table_1.docx]

Supplementary Material 1: Topic Categories

**Supplementary Material 1**: Overview of the 20 synthesized interest topics provided by laypersons in PLan Psy Study I, their respective content and relative frequency.

| **Topic** | **Content** | **Frequency** (N_total_= 1718) | **Relative Frequency** (%) |
| --- | --- | --- | --- |
| Developmental Psychology | Development during childhood & adolescence; parenting;  generational & cultural comparisons | 211 | 12.28 |
| Experimental Psychology, Neuropsychology, Biopsychology | Sensory perception; thought & memory processes; brain & neuro-research | 166 | 9.66 |
| Clinical Psychology: Depression | Types of depression; causes of depression; treatment of depression | 130 | 7.57 |
| General Social Psychology | Human interaction; interpersonal influence in groups; attitude change | 129 | 7.51 |
| Health Psychology | Interplay of body and mind on well-being; health behavior promotion; resilience | 113 | 6.58 |
| General Clinical Psychology & Other Disorders | Disorders unmentioned in other categories; suicidality; therapy schools | 108 | 6.29 |
| Clinical Psychology: Stress & Stress Coping | Causes of stress, stress management, consequences of stress | 106 | 6.17 |
| Sexuality & Relationships | Couple relationships; gender differences; sexuality | 95 | 5.53 |
| Clinical Psychology: Neuroses & Anxiety Disorders | Anxiety disorder; panic attacks; obsessions | 95 | 5.53 |
| Personality Psychology | Character traits; interindividual differences; self-worth and self-actualization | 90 | 5.24 |
| Society & Current World Issues | COVID-19; migration & immigration; environment & climate change | 66 | 3.84 |
| Industrial & Organizational Psychology & Consumer Psychology | Work satisfaction; coaching & leadership styles; sales strategies | 64 | 3.73 |
| Clinical Psychology: Neurological & Somatic Disorders | Alzheimer's disease; ADHD; physical illness caused by psychological factors | 61 | 3.55 |
| Communication & Media Psychology | Conflict management; online communication; digital media consumption | 51 | 2.97 |
| Clinical Psychology: Trauma | Consequences of trauma; consequences of abuse; Post-Traumatic Stress Disorder | 50 | 2.91 |
| Clinical Psychology: Addiction | Use & abuse of substances; controlled drinking; impact of abuse on the social environment | 47 | 2.74 |
| Educational Psychology | Early interventions & learning processes; intellectual giftedness; classroom teaching | 44 | 2.56 |
| Clinical Psychology: Personality Disorders | Types of personality disorders; Borderline Personality Disorder; self-harming behavior | 36 | 2.10 |
| Clinical Psychology: Psychodynamics | Psychoanalysis; depth psychology; dream interpretation | 29 | 1.67 |
| Forensic Psychology | Forensics; criminal profiling; causes of criminal behavior | 27 | 1.67 |

*Note:* Content = topic content covered by the respective category; Frequency = number of answers referring to topics from the respective category; N_total_ = amount of all answers provided in Study I; Relative Frequency = percentage of the respective topic category with reference to N_total_ in Study I.
