# Supplementary material for: To Have the Best Interest at Heart: Analyzing the Match Between Laypersons’ Interests and Publication Activity in Psychology
Source: Front Psychol. 2022 Jun 2;13:899430. doi: 10.3389/fpsyg.2022.899430 (PMC9201961; doi:10.3389/fpsyg.2022.899430)
Supplement: Supplementary Material 2 — Overview of all search query codes from PsycInfo and PSYNDEX for replication. [file Table_2.docx]

Supplementary Material 2: Search Queries

**Supplementary Material 2:** Overview of all search query codes from PsycInfo and PSYNDEX for replication.

| **Topic** | **Query Code PsycInfo (OVID)** | **Query Code PSYNDEX (PubPsych)** |
| --- | --- | --- |
| Clinical Psychology: Stress & Stress Coping | (stress* or burnout*).ti. not posttraumatic*.ti,sh. | ((TI=stress* or burnout*) not  (CT="posttraumatic stress disorder" or TI=("posttraumatic*" or PTSD or posttraumatisch* or PTBS))) |
| Clinical Psychology: Depression | 3211.cc. or (depress* or bipolar*).ti. | (SH=3211 or TI=(depress* or bipolar*)) |
| Personality Psychology | (3100 or 3120 or 3140).cc. | (SH=(3100 or 3120 or 3140)) |
| Health Psychology | (3120.cc. and (health* or "life satisfaction").hw,ti.)  or 3365.cc. or (resilien* or "positive psychology").ti,hw. | (SH=3130 and (CT=(health* or gesundheit* or  "life satisfaction" or lebenszufriedenheit) or TI=(health* or gesundheit* or "life satisfaction" or lebenszufriedenheit)) or SH=3365 or CT=(resilien* or "positive psychology") or TI=(resilien* or "positive psychology" or "positive psychologie")) |
| General Social Psychology | 30*.cc. | (SH=30*) |
| Clinical Psychology: Neurological & Somatic Disorders | (3250 or 3290 or 3293 or 3297).cc.  not stress*.ti,hw. | (SH=(3250 or 3290 or 3293 or 3297) not  (TI=stress* or CT=stress*)) |
| Experimental Psychology, Neuropsychology, Biopsychology | ((2300 or 232* or 233* or 234* or 236* or 2500 or 2510 or 2520 or 2540).cc. or (2530.cc. and "cognitive processes".sh.) or (2560.cc. not stress*.hw.)) not 32*.cc. | ((SH=(2300 or 232* or 233* or 234* or 236* or 2500 or 2510 or 2520 or 2540) or (SH=2530 and CT="cognitive processes") or (SH=2560 not CT=stress)) not SH=32*) |
| Clinical Psychology: Neuroses & Anxiety Disorder | 3215.cc. or (anxiet* or compulsi* or panic* or hypervigilan*).ti. not (trauma* or posttraumatic*).hw | (((SH=3215 or TI=(anxiet* or "compulsi*" or panic* or hypervigilan* or angst* or zwang* or panik*)) not CT=(trauma* or posttraumatic*))) |
| Communication & Media Psychology | (2700 or 2750).cc. or ("verbal communication" or "nonverbal communication" or "conflict resolution" or "social media" or "online social networks" or "media consumption").ti,sh. or (3020.cc. and "interpersonal communication".ti,sh.) | (SH=(2700 or 2750) or CT=("verbal communication" or "nonverbal communication" or "conflict resolution" or "social media" or "online social networks") or TI=("verbal communication" or "nonverbal communication" or "conflict resolution" or "social media" or "online social networks" or medienkonsum* or konfliktbewältigung*) or (SH=3020 and (CT="interpersonal communication" or TI=("interpersonal communication" or "zwischenmenschliche kommunikation" or "interpersonale kommunikation")))) |
| Clinical Psychology: Personality Disorders | 3217.cc. | (SH=3217) |
| Developmental Psychology | (28* or 2956).cc. | (SH=(28* or 2956)) |
| Forensic Psychology | (3236 or 3386 or 42*).cc. | (SH=(3236 or 3386 or 42*)) |
| Society & Current World Issues |  |  |
| Coronavirus | (corona or coronavirus or covid-19 or sars-cov-2).ti,sh. | (TI=(corona or coronavirus or covid-19 or  sars-cov-2) or CT=(coronavirus or covid-19)) |
| Lockdown & Qurantine | (lockdown or quarantine).ti,sh. | (TI=(lockdown or quarantine or quarantäne)  or CT=quarantine) |
| Climate Change | ("climatic change" or "climate change"  or "global warming").ti,sh. | (TI=(klimawandel or "globale erwärmung" or "climatic change" or "climate change" or "global warming") or CT=("climate change" or "global warming")) |
| Migration | (migration or migrant* or immigration or immigrant* or asylum* or refugee*).ti,hw. | (TI=(migration or migrant* or immigration or immigrant* or asylum* or refugee* or zuwanderung or einwanderung or flucht* or geflüchtet* or flüchtling*) or CT=("human migration" or immigration or "asylum seeking" or refugees)) |
| Political Crises | 2960.cc. and (crisis or crises).ti,hw. | (SH=2960 and (TI=(crisis or crises or krise*) or CT=(crisis or crises))) |
| Sexuality & Relationships | (2970 or 2980 or 2950).cc. or (paraphil* or "sexual development" or "psychosexual development").ti,sh. | (SH=(2970 or 2980 or 2950) or TI=(paraphil* or "sexual development" or "psychosexual development" or "sexuelle Entwicklung" or "Sexualentwicklung") or CT=("sexual development" or "psychosexual development" or paraphilias)) |
| Clinical Psychology: Trauma | (trauma or "child abuse" or "emotional abuse" or "verbal abuse" or "sexual abuse").ti,hw. | ((TI=trauma* or "child abuse" or "emotional abuse" or "verbal abuse" or "sexual abuse") or CT=(trauma or "child abuse" or "emotional abuse" or "verbal abuse" or "sexual abuse") or TI=(missbrauch not drogen)) |
| Clinical Psychology: Addiction | (3233 or 3383).cc. | (SH=(3233 or 3383)) |
| General Clinical Psychology & Other Disorders | (3213 or 3260 or 3230 or 33*).cc. Not (3315 or 3365 or 3383 or 3386 or 3250 or 3290 or 3293 or 3297 or 3211 or 3215 or 3217 or 3233).cc. | (SH=(3213 or 3260 or 3230 or 33*) not (3315 or 3365 or 3383 or 3386 or 3250 or 3290 or 3293 or 3297 or 3211 or 3215 or 3217 or 3233)) |
| Clinical Psychology: Psychodynamics | (3143 or 3315).cc. | (SH=(3143 or 3315)) |
| Educational Psychology | 35*.cc. | (SH=35*) |
| Industrial & Organizational Psychology & Consumer Psychology | (36* or 39*).cc. or (4030.cc. and "work space".sh,ti.) | (SH=(36* or 39*) or (SH=4030 and (CT="work space" or TI=("work space" or "arbeitsplatz")))) |

*Note:* The category “Society & Current World Issues” did not receive individual *PsycInfo* or *PSYNDEX* query codes since searches for it were composed of a variety of different sub-topics (i.e., “Coronavirus” or “Climate Change”).
